# Space-time analysis of work-related musculoskeletal disorders in Brazil: an ecological study

**DOI:** 10.1590/0102-311XEN141823

**Published:** 2024-07-22

**Authors:** Alanna Gleice Carvalho Fontes Lima, Caíque Jordan Nunes Ribeiro, Shirley Verônica Melo Almeida Lima, Yanna Menezes Barbosa, Iris Machado de Oliveira, Karina Conceição Gomes Machado de Araújo

**Affiliations:** 1 Universidade Federal de Sergipe, Aracaju, Brasil.; 2 Universidad de Vigo, Pontevedra, España.

**Keywords:** Time Series Studies, Spatial Analysis, Occupational Health, Musculoskeletal Diseases, Estudos de Séries Temporais, Análise Espacial, Saúde Ocupacional, Doenças Musculoesqueléticas, Estudios de Series Temporales, Análisis Espacial, Salud Laboral, Enfermedades Musculoesqueléticas

## Abstract

This study aimed to analyze the incidence of work-related musculoskeletal disorders (WMSD) in Brazil from 2007 to 2019, examining the spatial, temporal, and spatiotemporal patterns of their occurrence. An ecological time series study was conducted using spatial analysis techniques. WMSD morbidity data from 2007 to 2019 were collected from the Brazilian Information System for Notificable Diseases of the Brazilian Health Informatics Department. Incidence rates were standardized and smoothed using the local empirical Bayes’ theorem. Time trends were analyzed by segmented linear regression. Spatial analysis was performed using Moran’s univariate global (I) and local (LISA) indexes. The spatiotemporal scan statistic was used to identify high-risk spatiotemporal clusters for WMSD. A total of 93,387 cases of WMSD were recorded in Brazil. Temporal trends showed an increase in all regions except the Northeast, which remained stable. The incidence of WMSD showed a spatial dependence, with spatial and space-time clusters identified, especially in the Southeast region, overlapping the largest economic-industrial center of the country. The spatiotemporal clustering observed in one region suggests the highest level of industrial and economic development. Our findings highlight the need to implement intersectoral surveillance policies, inspect working conditions, and invest in the prevention and promotion of workers’ health.

## Resumo

O objetivo foi analisar a incidência de distúrbios osteomusculares relacionados ao trabalho (DORT) no Brasil de 2007 a 2019, examinando os padrões espaciais, temporais e espaço-temporais de sua ocorrência. Foi realizado um estudo ecológico de séries temporais utilizando técnicas de análise espacial. Os dados de morbidade por DORT de 2007 a 2019 foram coletados do Sistema de Informação de Agravos de Notificação do Departamento de Informática do SUS. As taxas de incidência foram padronizadas e suavizadas pelo método de Bayes empírico local. As tendências temporais foram examinadas por meio de regressão linear segmentada. A análise espacial foi realizada utilizando-se os índices univariados de Moran global (I) e índice de Moran local (LISA). A estatística de varredura espaço-temporal foi aplicada para identificar aglomerados espaço-temporais de DORT de alto risco. Foram registrados 93.387 casos de DORT no Brasil. A tendência temporal mostrou aumento em todas as regiões, exceto no Nordeste, que se manteve estável. A incidência de DORT apresentou dependência espacial e foram identificados aglomerados espaciais e espaço-temporais, particularmente na Região Sudeste, sobrepondo-se ao maior centro econômico-industrial do país. A aglomeração espaço-temporal observada em uma região sugere maior nível de desenvolvimento industrial e econômico. Os achados evidenciam a necessidade de implementação de políticas intersetoriais de vigilância, fiscalização das condições de trabalho e investimentos na prevenção e promoção da saúde do trabalhador.

## Resumen

El objetivo fue analizar la incidencia de los trastornos musculoesqueléticos relacionados con el trabajo (TMERT) en Brasil del 2007 al 2019, examinando los patrones espaciales, temporales y espacio-temporales de su incidencia. Se realizó un estudio ecológico de series temporales usando técnicas de análisis espacial. Los datos de morbilidad por TMERT del 2007 al 2019 se recolectaron del Sistema de Información de Enfermedades de Notificación Obligatoria del Departamento de Informática del Sistema Único de Salud. Las tasas de incidencia se estandarizaron y se suavizaron usando el método de Bayes empírico local. Las tendencias temporales se examinaron mediante regresión lineal segmentada. El análisis espacial se realizó utilizando los índices univariados de Moran global (I) y el índice de Moran local (LISA). Se aplicó la estadística de análisis espacio-temporal para identificar aglomerados espacio-temporales de TMERT de alto riesgo. En Brasil se registraron 93.387 casos de TMERT. La tendencia temporal mostró aumento en todas las regiones, salvo en el Nordeste, que se mantuvo estable. La incidencia de TMERT presentó dependencia espacial y se identificaron aglomerados espaciales y espacio-temporales, particularmente en la región Sudeste, superponiéndose al centro económico-industrial más grande del país. La aglomeración espacio-temporal observada en una región sugiere un mayor nivel de desarrollo industrial y económico. Los hallazgos resaltan la necesidad de implementar políticas intersectoriales de vigilancia, inspección de las condiciones de trabajo e inversiones en la prevención y promoción de la salud del trabajador.

## Introduction

Work-related musculoskeletal disorders (WMSD) are a significant occupational health problem that can reduce the functional capacity and quality of life of workers worldwide ^1^ ^,^ ^2^. Despite the prevalence of WMSD, epidemiological studies on WMSD are limited in the literature, making it difficult to identify their spatial patterns and estimate their true social and economic impact, especially in developing countries such as Brazil.

WMSD represent a silent epidemic that can lead to adverse outcomes for workers, entrepreneurs, and society as a whole. WMSD often affect workers in their productive years, leading to the need for medical treatment, time off work, and even disability retirement. Moreover, WMSD remain a neglected condition, especially in developing countries such as Brazil ^3^ ^,^ ^4^.

In the first quarter of 2020, Brazil’s working population was estimated to be 92.2 million people, of which 66.9% had an employment relationship, including domestic workers. In addition, 26.2% of these people worked without a formal contract, 4.8% were employers, and 2.1% were workers’ relatives or assistants. The North (33.6%) and Northeast (29.8%) regions had a higher percentage of autonomous workers than other regions of the country ^5^.

The last *Brazilian National Health Survey*, conducted in 2013, estimated that approximately 4,948,000 people aged 18 and over reported having suffered an accident at work in the previous 12 months. In comparison, only 717,911 occupational accidents were registered with the Social Security in 2013. These data show that almost seven times more people reported having suffered an accident at work compared with the accidents at work registered with the Social Security ^6^.

From 2007 to 2018, over 67,000 workers in Brazil reported suffering from WMSD. These disorders were found to be more prevalent among female workers (51.7%), those aged 40-49 years, and those with complete secondary education (32.7%). The Southeast region accounted for the highest number of cases during this period, with 58.4%, and the states with the highest incidence rates were Amazonas, Mato Grosso do Sul, and São Paulo ^7^.

Typical signs and symptoms of WMSD include pain, muscle weakness, paresthesia, and limited range of motion. As a result, affected workers are at increased risk of developing mental health disorders such as anxiety and depression. This cooccurrence of health problems can further delay return to work activities and prolong the impact of the disorder on the worker’s well-being ^8^ ^,^ ^9^.

Despite the importance of WMSD, there are few studies analyzing the epidemiology of these and other health problems among workers ^10^. Moreover, to date, no research has evaluated the spatial, temporal, and spatiotemporal patterns and magnitude of WMSD in Brazil. Therefore, this study aimed to analyze the incidence of WMSD in Brazil from 2007 to 2019, examining the spatial, temporal, and spatiotemporal patterns of their occurrence.

## Method

### Study design

This was an ecological time series study that used spatial, temporal, and spatiotemporal analysis techniques and included all confirmed cases of WMSD in Brazil from 2007 to 2019.

### Ethical considerations

This study complied with national ethical recommendations and the *Declaration of Helsinki*. The data used were aggregated, anonymous, and publicly available, so the study did not require the use of an informed consent form. The research project was approved by the Research Ethics Committee of the Federal University of Sergipe (registration n. 4,465,618).

### Study area description

The units of analysis of this study were the 5,568 municipalities of Brazil. Brazil is the largest country in Latin America, bordering the Atlantic Ocean to the east, with a coastline of 7,491km, and sharing borders with all other South American countries except Chile and Ecuador. The country is divided into five regions (North, Northeast, Southeast, South, and Central-West) and 27 Federative Units.

Despite its status as the world’s 9th largest economy, with a gross domestic product of BRL 8.7 trillion, Brazil remains one of the most unequal countries in the world. Its population is estimated at 207.7 million, with 51% of inhabitants being economically active ^11^. Brazil has an Human Development Index of 0.710, a per capita income of BRL 1,353.00, and a Gini index of 0.481. The country’s unemployment rate is 9.8%, and more than 38 million people are in the informal labor market.

### Data sources

WMSD morbidity data from 2007 to 2019 were collected from the Braziliam Information System for Notificable Diseases (SINAN, acronym in Portuguese) of the Brazilian Health Informatics Department (DATASUS, acronym in Portuguese) ^12^. Population estimates for the intercensal years were derived from the 2000 and 2010 censuses, and digital cartographic bases for Brazil (shapefile extension), presented in the latitude/longitude system (SIRGAS 2000), were obtained from the Brazilian Institute of Geography and Statistics (IBGE, acronym in Portuguese) ^5^.

### Variables and measures

The variables analyzed in this study were year of occurrence, demographics (self-reported sex/gender, age group, race/skin color, residence zone, and education level), case type, outcome, comorbidities, and clinical manifestations ( Supplementary Material - Table S1 https://cadernos.ensp.fiocruz.br/static//arquivo/suppl-e00141823_6851.pdf). The dependent variable of this study was the mean incidence rate of WMSD at the municipal level. This rate was calculated by dividing the average of new WMSD cases by the working age population of the median year of the time series (2013), multiplied by a constant of 100,000.

The working age population refers to the age group of individuals who are capable of engaging in economic activity. While the age range for this classification varies between countries, in this study, a lower limit of 10 years of age was used, as child labor is still prevalent in Brazil ^13^.

### Temporal trend analysis

The temporal trend analysis was conducted using segmented linear regression (joinpoint). This method made it possible to identify changes in the trend of WMSD incidence over time by fitting data from a series with the smallest possible number of joinpoint ^14^.

The dependent variable of the model was the Neperian logarithm of the crude incidence rate of WMSD by sex, age group, and region, while the independent variable was the year of the time series. Segmented linear regression (joinpoint) was used to analyze the temporal trend. Annual percentage changes (APC) were calculated for each segment, and average annual percentage changes (AAPC) were calculated for the entire period when there was more than one significant inflection point, along with their respective 95% confidence intervals (95%CI).

APC and AAPC were considered significant if the p-values were less than 0.05 and the 95%CI did not include the value zero. The final model was the best fitted and best represented the trend with the least number of inflection points ^15^.

When interpreting the results, significant and positive APC/AAPC were considered to indicate an upward trend, whereas negative and significant APC/AAPC were considered to indicate a downward trend. If no statistical significance was found, the trend was considered stable, regardless of the value of the APC/AAPC ^16^.

### Spatial analysis

The first stage of the spatial analysis involved a descriptive analysis that mapped the distribution of crude and smoothed mean rates of WMSD incidence. Rate smoothing was performed using the local empirical Bayesian method to reduce random rate fluctuation in municipalities with small populations ^17^.

The second stage of spatial analysis aimed to identify the presence of spatial dependence and was performed using the univariate global Moran’s index (I). This index ranges from -1 to +1, with values close to zero indicating spatial randomness, positive values indicating positive spatial autocorrelation, and negative values indicating negative spatial autocorrelation ^18^.

The final stage of spatial analysis aimed to identify spatial clusters by calculating the local indicators of spatial association (LISA) Moran’s index, which generated a scatterplot with four quadrants: Q1 (high/high or hotspots: positive values, positive means), Q2 (low/low or cold spots: negative values, negative means), Q3 (high/low: positive values, negative means), and Q4 (low/high: negative values, positive means) ^18^.

The results of the analysis were represented spatially using Moran maps, which only included municipalities with statistically significant local indicators (p < 0.05) ^18^.

### Space-time analysis

Kulldorf’s retrospective space-time scan analysis was used to detect high-risk clusters for WMSD. This method made it possible to identify simultaneous spatial and temporal proximity between cases based on the numbers recorded by the municipality of residence and the population estimate for the study period ^19^.

The discrete Poisson probability model was chosen for the scan, assuming the null hypothesis that the number of expected cases in each area is proportional to the population size ^20^. Space-time modeling was carried out with the following parameters: one year aggregation time; no geographical or temporal overlapping of clusters; circular clusters; maximum spatial cluster size equal to 50% of the exposed population; and maximum temporal cluster size equal to 50% of the study period.

The primary and secondary clusters with the highest likelihood were identified using the log-likelihood ratio (LLR) test and presented in maps and tables. Relative risks (RR) for the incidence of work-related musculoskeletal pain were calculated for each cluster, considering neighboring areas. The statistical significance of the results was determined using 999 Monte Carlo simulations, and p-values < 0.05 were considered significant ^19^.

### Software

Data were stored and processed using Microsoft Office Excel 2016 (https://products.office.com/). Thematic maps were generated using QGIS 3.4.11 (https://qgis.org/en/site/), while spatial analysis was conducted in GeoDa 1.14 (https://spatial.uchicago.edu/geoda). Time trends were analyzed using Joint Point Regression 4.6 (https://surveillance.cancer.gov/joinpoint/), and SaTScan 9.6 (http://www.satscan.org) was used for spatiotemporal cluster analysis.

## Results

During the analysis period ₋ from 2007 to 2019 ₋, a total of 93,387 WMSD cases were reported in the 5,568 municipalities of Brazil. However, three cases were excluded from the analysis due to lack of documented municipal allocation. The Southeast region had the highest number of recorded cases during the analyzed period, with 57.1% (53,336) of the notifications, followed by the Northeast region, with 25.95% (24,234). In contrast, the North region had the lowest number of recorded cases, representing only 3.16% (2,948) of the total.

The majority of the workers with WMSD in Brazil were women (48,702; 52.15%), aged 25 to 44 years (49,457; 52.95%), had completed basic education (< 12 years of schooling) (59,926; 64.17%), were white (36,865; 39.48%), lived in urban areas (83,741; 89.67%), and were formally employed (61,793; 66.17%) (Table 1).

**Table 1:** Sociodemographic characteristics of the study population. Brazil, 2007-2019.

Characteristics	North (n = 2,948)		Notrheast (n = 24,234)		Southeast (n = 53,336)		South (n = 9,711)		Central-West (n = 3,155)		Brazil (n = 93,384)	
	n	%	n	%	n	%	n	%	n	%	n	%
Sex												
Male	1,163	39.45	11,191	46.18	27,621	51.79	3,743	38.54	955	30.27	44,673	47.84
Female	1,785	60.55	13,041	53.81	25,710	48.20	5,966	61.44	2,200	69.73	48,702	52.15
Unknown/Not applicable/Missing	-	-	2	0.01	5	0.01	2	0.02	-	-	9	0.01
Age (years)												
< 15	-	-	5	0.02	7	0.01	-	-	1	0.03	13	0.01
15-24	140	4.75	1,001	4.13	2,447	4.59	418	4.30	106	3.36	4,112	4.40
25-44	1,962	66.55	13,558	55.95	28,324	53.10	4,272	43.99	1,331	42.19	49,447	52.95
45-59	780	26.46	8,977	37.04	20,319	38.10	4,331	44.60	1,433	45.42	35,840	38.38
≥ 60	66	2.24	693	2.86	2,239	4.20	690	7.11	284	9.00	3,972	4.25
Schooling												
No education	17	0.58	288	1.19	315	0.59	67	0.69	13	0.41	700	0.75
Basic education	2,142	72.66	15,649	64.57	34,297	64.30	6,197	63.81	1,641	52.01	59,926	64.17
Higher education	503	17.06	3,402	14.04	3,608	6.76	486	5.00	332	10.52	8,331	8.92
Unknown/Not applicable/Missing	286	9.70	4,895	20.19	15,116	28.34	2961	30.49	1,169	37.05	24,427	26.15
Ethnicity/Skin color												
White	631	21.40	3,868	15.96	24,421	45.79	6,619	68.16	1,326	42.03	36,865	39.48
Non-white	2,232	75.71	14,706	60.68	13,421	25.16	1,221	12.57	1,295	41.05	32,875	35.20
Unknown/Not applicable/Missing	85	2.89	5,660	23.36	15,494	29.05	1871	19.27	534	16.92	23,662	25.34
Zone of residence												
Urban	2,811	95.35	21,571	89.01	48,296	90.55	8,222	84.67	2,841	90.05	83,741	89.67
Rural	61	2.07	1,119	4.62	1,583	2.97	859	8.85	69	2.19	3,691	3.95
Periurban	4	0.14	100	0.41	144	0.27	75	0.77	6	0.19	329	0.35
Unknown/Not applicable/Missing	72	2.44	1444	5.96	3313	6.22	555	5.72	239	7.57	5623	6.02
Employment status												
Formally registered employee	1,402	47.56	15,704	64.80	37,252	69.84	5,869	60.44	1,566	49.64	61,793	66.17
Informal employee	66	2.24	362	1.49	742	1.39	139	1.43	84	2.66	1,393	1.49
Public servant	439	14.89	1,987	8.20	2,258	4.23	586	6.03	630	19.97	5,900	6.32
Temporary, independent, cooperative, or self-employed worker	224	7.60	1,701	7.02	3,885	7.28	1,472	15.16	503	15.94	7,785	8.34
Unemployed	640	21.71	2,943	12.14	2,865	5.37	609	6.27	135	4.28	7,192	7.70
Retired	18	0.61	171	0.71	294	0.55	150	1.54	78	2.47	711	0.76
Unknown/Not applicable/Missing	159	5.39	1366	5.64	6,040	11.33	886	9.12	159	5.04	8,610	9.22

The most common clinical characteristics observed in the population were pain (77,246; 82.72%), followed by task limitation (70,270; 75.25%), movement limitation (55,781; 63.33%), decrease in muscle strength (55,781; 59.73%), and the need to take time off work for treatment, in over half of the cases (54,661; 58.53%). In addition, temporary disability was the most frequent outcome, accounting for 53.46% (49,923) of the cases (Table 2).

**Table 2:** Clinical characteristics of the study population. Brazil, 2007-2019.

Characteristics	North (n = 2,948)		Northeast (n = 24,234)		Southeast (n = 53,336)		South (n = 9,711)		Cental-West (n = 3,155)		Brazil (n = 93,384)	
	n	%	n	%	n	%	n	%	n	%	n	%
Hypertension												
Yes	334	11.33	3,992	16.47	5,701	10.69	1,536	15.82	662	20.98	12,225	13.09
No	2,324	78.83	15,438	63.70	29,768	55.81	6,330	65.18	1,796	56.93	55,656	59.60
Unknown/Not applicable/Missing	290	9.84	4,804	19.82	17,867	33.50	1,845	18.99	697	22.10	25,503	27.31
Diabetes												
Yes	99	3.36	859	3.54	1,478	2.77	462	4.76	171	5.42	3,069	3.29
No	2,557	86.74	18,584	76.69	33,640	63.07	7,365	75.84	2,227	70.59	64,373	68.93
Unknown/Not applicable/Missing	292	9.90	4,791	19.77	18,218	34.15	1,884	19.40	757	24.00	25,942	27.78
Mental disorders												
Yes	82	2.78	956	3.94	1,287	2.41	509	5.24	84	2.66	2,918	3.12
No	2,261	76.70	17,302	71.40	33,585	62.97	7,237	74.52	2,290	72.58	62,675	67.12
Unknown/Not applicable/Missing	605	20.52	5,976	24.66	18,464	34.62	1,965	20.23	781	24.75	27,791	29.76
Sensitivity alteration												
Yes	1,681	57.02	11,962	49.36	15,310	28.70	3,891	40.07	1,404	44.50	34,248	36.67
No	995	33.75	8,117	33.49	18,953	35.54	4,266	43.93	1,168	37.02	33,499	35.87
Unknown/Not applicable/Missing	272	9.23	4,155	17.14	19,073	35.76	1,554	16.00	583	18.48	25,637	27.46
Movement limitation												
Yes	2,179	73.91	17,166	70.83	30,918	57.97	6,633	68.30	2,248	71.25	59,144	63.33
No	544	18.45	3,883	16.02	8,986	16.85	1,812	18.66	536	16.99	15,761	16.88
Unknown/Not applicable/Missing	225	7.63	3,185	13.14	13,432	25.19	1,266	13.04	371	11.76	18,479	19.79
Decresead muscle strength												
Yes	2,134	72.39	16,140	66.60	25,525	47.86	5,673	58.42	2,123	67.29	51,595	55.25
No	575	19.50	4,922	20.31	12,027	22.55	2,626	27.04	535	16.96	20,685	22.15
Unknown/Not applicable/Missing	239	8.10	3,172	13.08	15,784	29.60	1,412	14.54	497	15.75	21,104	22.60
Decreased movement												
Yes	2,042	69.27	16,356	67.49	29,343	55.02	6,000	61.79	2,040	64.66	55,781	59.73
No	661	22.42	4,611	19.03	9,452	17.72	2,380	24.51	648	20.54	17,752	19.01
Unknown/Not applicable/Missing	245	8.31	3,267	13.48	14,541	27.27	1,331	13.70	467	14.80	19,851	21.25
Phlogisitic signs												
Yes	1,244	42.20	5,881	24.27	10,802	20.25	2,302	23.71	964	30.55	21,193	22.69
No	1,243	42.16	12,837	52.97	22,026	41.30	5,552	57.17	1,484	47.04	43,142	46.20
Unknown/Not applicable/Missing	461	15.63	5,516	22.77	20,508	38.45	1,857	19.12	707	22.41	29,049	31.11
Pain												
Yes	2,703	91.69	21,797	89.94	41,463	77.74	8,461	87.13	2,822	89.45	77,246	82.72
No	63	2.14	454	1.87	853	1.60	213	2.19	86	2.73	1,669	1.79
Unknown/Not applicable/Missing	182	6.17	1,983	8.18	11,020	20.66	1037	10.68	247	7.83	14,469	15.49
Task limitations												
Yes	2,235	75.81	20,049	82.73	38,137	71.50	7,366	75.85	2,483	78.70	70,270	75.25
No	403	13.67	1,356	5.60	5,958	11.17	1,273	13.11	408	12.93	9,398	10.06
Unknown/Not applicable/Missing	310	10.52	2,829	11.67	9,241	17.33	1,072	11.04	264	8.36	13,716	14.68
Abscence for treatment												
Yes	1,744	59.16	16,114	66.49	30,086	56.41	4,971	51.19	1,746	55.34	54,661	58.53
No	897	30.43	5,038	20.79	12,714	23.84	3,126	32.19	806	25.55	22,581	24.18
Unknown/Not applicable/Missing	307	10.41	3,082	12.72	10,536	19.75	1,614	16.62	603	19.11	16,142	17.29
Outcomes												
Temporary disability	970	32.90	15,822	65.29	26,559	49.80	4,833	49.77	1,739	55.12	49,923	53.46
Permanent partial disability	362	12.28	1,837	7.58	2,072	3.88	624	6.43	207	6.56	5,102	5.46
Total permanent disability	50	1.70	206	0.85	128	0.24	40	0.41	17	0.54	441	0.47
Death	2	0.07	3	0.01	7	0.01	5	0.05	3	0.10	20	0.02
Cure	383	12.99	1,536	6.34	5,805	10.88	1,125	11.58	263	8.34	9,112	9.76
Unknown/Not applicable/Missing	1,181	40.06	4,830	19.93	18,765	35.19	3,084	31.75	926	29.35	28,786	30.82

**Figure 1:**
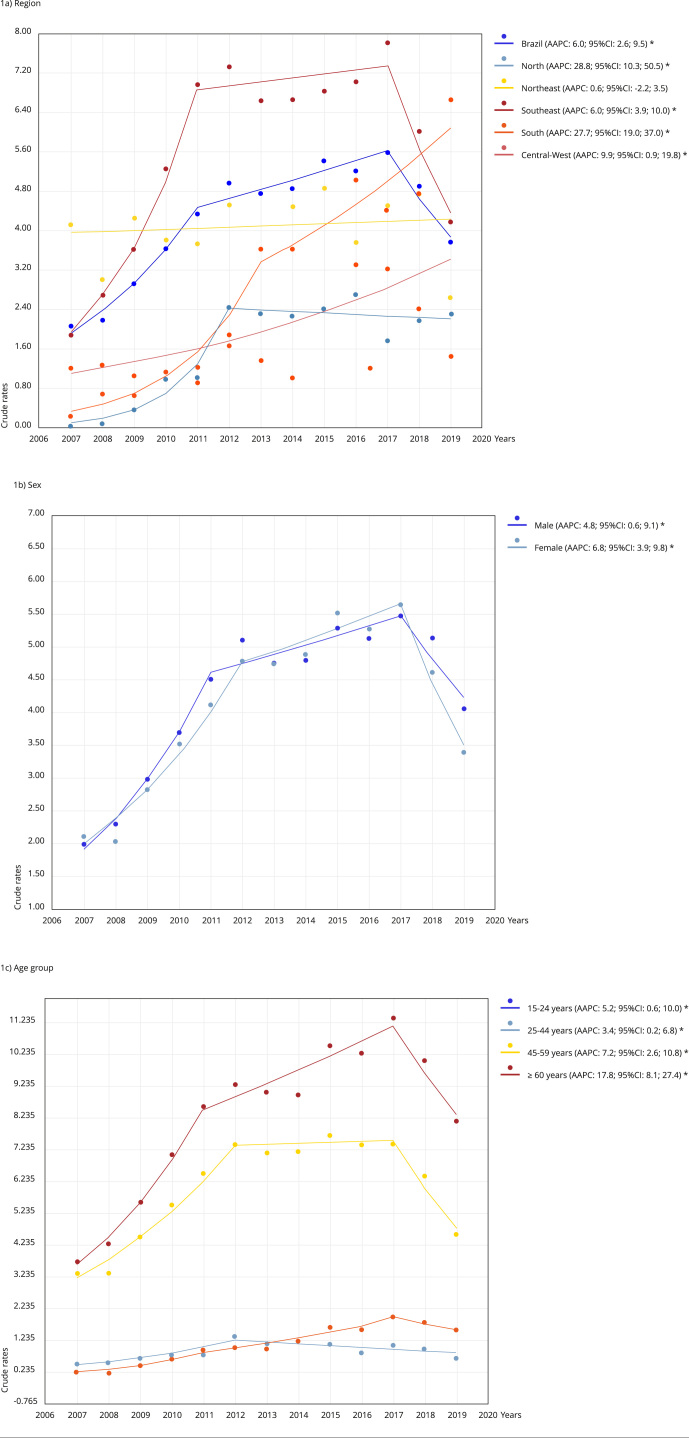
Temporal trend analysis of work-related musculoskeletal disorders incidence by region, sex, and age group. Brazil, 2007-2019.

Figure 1 shows the average trends in crude incidence rates of WMSD cases by region (1a), sex (1b), and age group (1c). The highest percentage increases were observed in the North (AAPC: 28.8%) and South (AAPC: 27.7%) regions, particularly among women (AAPC: 6.8%) and older adults (AAPC: 17.8%). Table 3 provides more detailed information on the trend segments, among which there was a significant increase from 2007 to 2011 throughout the country. Overall, the temporal trends increased in all regions except the Northeast, where the trends remained stable. The North and South regions, as well as women and older adults, had the highest percentage increases.

95%CI: 95% confidence interval; AAPC: average annual percentage change. * p-value < 0.05.

**Table 3:** Time trend analysis of work-related musculoskeletal disorders by region, sex, and age group. Brazil, 2007-2019.

Indicator	Segmented period		
	Period	APC (95%CI)	Trend
Area			
Brazil	2007-2011	23.6 (14.8; 33.1) *	Upward
	2011-2017	3.9 (0.1; 7.8) *	Upward
	2017-2019	-17.1 (-30.4; -1.2) *	Downward
North	2007-2012	86.9 (21.8; 186.6) *	Upward
	2012-2019	-1.3 (-7.9; 5.9)	Stable
Northeast	2007-2019	0.6 (-2.2; 3.5)	Stable
Southeast	2007-2011	36.9 (28.2; 46.3) *	Upward
	2011-2017	1.1 (-1.8; 4.2)	Stable
	2017-2019	-22.9 (-34.1; -9.9) *	Downward
South	2007-2013	47.7 (26.6; 72.4) *	Upward
	2013-2019	10.4 (4.0; 17.1) *	Upward
Cental-West	2007-2019	9.9 (0.9; 19.8) *	Upward
Sex			
Male	2007-2012	19.1 (11.8; 26.8) *	Upward
	2012-2017	3.5 (-3.0; 10.4)	Stable
	2017-2019	-21.3 (-37.3; -1.3)	Downward
Female	2007-2012	24.4 (16.6; 32.7) *	Upward
	2012-2017	2.9 (-0.3; 6.2)	Stable
	2017-2019	-12.1 (-24.2; 1.8)	Stable
Age (years)			
15-24	2007-2012	21.7 (9.8; 35.0) *	Upward
	2012-2019	-5.3 (-10.0; -0.3) *	Downward
25-44	2007-2012	18.1 (12.7; 23.8) *	Upward
	2012-2017	0.4 (-4.5; 5.6)	Stable
	2017-2019	-20.1 (-33.5; -4.1)	Downward
45-59	2007-2012	23.7 (14.0; 34.2) *	Upward
	2012-2017	4.6 (0.6; 8.7) *	Upward
	2017-2019	-13.4 (-27.1; 2.8)	Stable
≥ 60	2007-2012	38.7 (8.2; 77.8) *	Upward
	2012-2017	14.6 (4.9; 25.2) *	Upward
	2017-2019	-9.7 (-34.1; 23.7)	Stable

95%CI: 95% confidence interval; APC: annual percentage change.

* p-value < 0.05.

**Figure 2:**
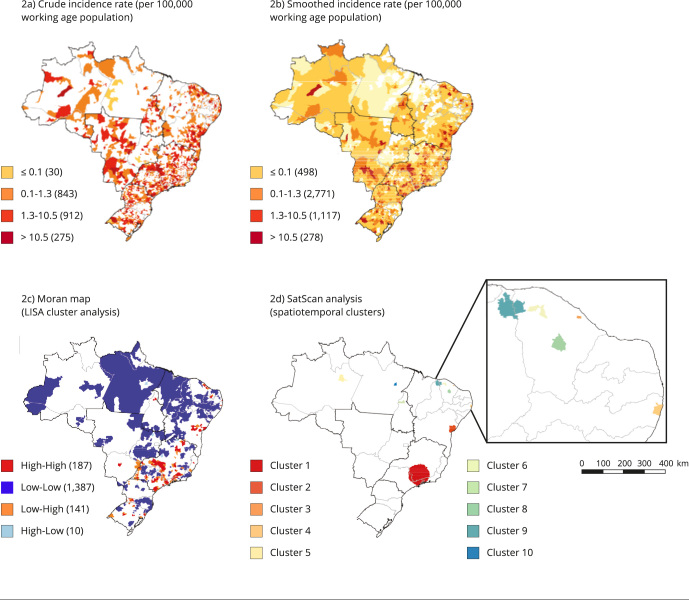
Spatial and spatiotemporal analysis of work-related musculoskeletal disorders incidence. Brazil, 2007-2019.

The spatial distribution of WMSD incidence is shown in the choropleth maps in Figure 2a, in which the highest density of crude incidence rates is concentrated in the Southeast region. The rates smoothed by the local empirical Bayesian method show a diffuse distribution between the Central-West and Southeast regions and the states of Paraná, Bahia, Ceará, and Rio Grande do Sul (Figure 2b).

Spatial autocorrelation analysis was performed by calculating the univariate global Moran’s index (I = 0.107; p = 0.001). The results were statistically significant, indicating the presence of spatial dependence between the occurrence of WMSD cases in municipalities with similar patterns.

Figure 2c shows the Moran map indicating the clusters of municipalities identified in the scatter diagram generated by the univariate LISA analysis. High-risk clusters were predominantly observed in the Southeast region and, to a lesser extent, in the Northeast region, specifically in the states of Ceará and Bahia.

Space-time analysis was performed using space-time scan statistics, which revealed 12 statistically significant space-time clusters of new WMSD cases in the working age population. These clusters are listed in Table 4 and visualized in Figure 2d.

**Table 4:** Spatiotemporal clusters of annual crude incidence rates of work-related musculoskeletal disorders (WMSD). Brazil, 2007-2019.

Cluster	Period	Municipalities	States	New cases	Expected cases	Annual incidence rate *	RR	LLR
1	2010-2015	380	Minas Gerais, Rio de Janeiro, São Paulo	12,991	2,507	21.9	5.9	11,217.7
2	2007-2012	46	Bahia	5,176	1,102	19.9	4.9	4,025.5
3	2009-2014	1	Ceará	540	12	187.5	44.5	1,520.7
4	2012-2017	9	Pernambuco	2,692	795	14.3	3.5	1,404.9
5	2012-2016	2	Amazonas	1,273	355	15.2	3.6	713.4
6	2011-2016	1	Ceará	357	43	35.3	8.4	443.8
7	2016-2019	1	Tocantins	284	25	47.8	11.3	430.0
8	2014-2019	1	Ceará	218	17	54.9	13.0	357.7
9	2010-2012	11	Piauí, Ceará	104	26	17.1	4.0	66.9
10	2019	1	Pará	35	4	37.6	8.9	45.4

LLR: likelihood ratio; RR: relative risk (for the cluster compared with the rest of the region).

* WMSD incidence rate (per 100,000 working age population) during the clustering period.

The primary cluster ₋ which had the highest number of new cases (380) from 2010 to 2015 ₋ was located in municipalities of three states in the Southeast region (Minas Gerais, São Paulo, and Rio de Janeiro). The gross annual incidence rate was 21.9/100,000, with a RR of 5.9 (p < 0.001). Notably, the cluster with the highest RR (44.5; p < 0.001) consisted of only one municipality in the Northeast region (state of Ceará). The high-risk spatiotemporal clustering pattern is similar to that obtained in the univariate LISA, as shown in the Moran map (Figure 2c).

## Discussion

This study is the first to provide a comprehensive overview of the epidemiology of WMSD in Brazil, covering the 13-year period from the beginning of compulsory notification until 2019. The unprecedented findings reported here are essential for understanding the spatial, temporal, and spatiotemporal patterns of these injuries and for revealing the magnitude of a health problem that partially or permanently disables thousands of Brazilian workers.

It is important to note that in Brazil, the National Policy for Workers’ Health was only implemented in 2012. However, the compulsory notification of WMSD was established by Ruling of the Brazilian Ministry of Health in 2004 and became effective in 2007 ^21^. Nevertheless, the essential principles of workers’ health are well established in Brazilian regulation, with a strong presence in the Organic Law of Health and in the regulatory norms published by the Brazilian Ministry of Labor and Employment.

In our study, the average annual incidence rate of 4.2 per 100,000 working-age inhabitants highlights the burden of WMSD on the Brazilian workforce. To the best of our knowledge, epidemiological studies on the prevalence and incidence of WMSD in Brazil are limited, and systematic reviews of observational studies are available only for specific occupational categories in specific regions of the world ^22^ ^,^ ^23^ ^,^ ^24^. Therefore, our study fills a critical gap in the knowledge of the epidemiology of WMSD in Brazil.

In Canada, the prevalence of WMSD among farmers was reported to be 85.6%. In Jimma, Ethiopia, the prevalence of WMSD among bank workers was 73.1% in 2019, while among nurses in Ibadan, Nigeria, WMSD were present in 84.4% of those screened. This high prevalence of WMSD highlights the silent epidemic occurring worldwide in a variety of occupations ^22^ ^,^ ^23^ ^,^ ^24^.

Although this study considered 10 years as the starting working age, it is important to note that only individuals aged 15 years and older had notifications officially registered on SINAN. These data highlight the fact that individuals who are more vulnerable to degrading working conditions may not be visible in this system, which could lead to an underestimation of the true prevalence of illegal child labor.

Regarding sex, more than half of the reported cases occurred among female workers. This finding is consistent with some studies that have reported a higher prevalence of WMSD among women ^25^ ^,^ ^26^. The phenomenon has been attributed to the “double burden” of women, who often engage in both work and household chores. As a result, women may be exposed to physical demands beyond their capacity, leading to muscle and joint strain.

In addition, when analyzing the temporal trend by sex, a greater percentage increase was observed in women. This trend may be related to the increased participation of women in the labor market. This assertion is supported by sex statistics from the IBGE, which show that, from 2012 to 2019, the rate of WMSD increased by 2.9 percentage points among women and decreased by 1 percentage point among men ^5^.

Note that the significant increase in records of new cases of WMSD from 2007 to 2011 may be due to the implementation of the SINAN notification form. Because Brazil is a large country, the increase in case detection may vary according to the capacity of local surveillance systems to investigate and confirm reported cases.

Low education level was an important social determinant in our study. This may be due to the higher prevalence of menial and repetitive tasks in the group of workers with lower education levels compared to those with higher education ^26^ ^,^ ^27^.

Adults aged 24 to 59 years accounted for more than 90% of the total cases, which is expected given that this age group comprises the working age population. Furthermore, the interaction between the duration of exposure to activities, wear and tear of anatomical structures, and aging increases the likelihood of developing WMSD ^28^.

It is worth noting that despite the smaller proportion of older adults in the labor market, the proportion of this group with WMSD has increased, as shown by the large percentage increase in the temporal trend analyzed in our study. This suggests that measures to prevent and manage WMSD should also consider the aging workforce.

Pain stood out as one of the most common symptoms associated with WMSD, affecting more than 80% of the study population. This is supported by several surveys of workers from a variety of occupational categories, including dentists, white- and blue-collar workers, nurses, bank officers, and poultry farmers ^29^ ^,^ ^30^ ^,^ ^31^ ^,^ ^32^ ^,^ ^33^.

Functional disability due to musculoskeletal pain has been shown to be a significant limiting factor, affecting workers’ productivity and quality of life. This disability is also directly associated with anxiety and depression, highlighting the importance of addressing and preventing WMSD in the workplace ^8^ ^,^ ^34^.

Research has shown that dental professionals who experience pain in different parts of the body have a 20% reduction in productivity ^31^. Similarly, nurses in a public hospital have reported a positive association between pain and disability ^35^. Therefore, WMSD have a significant impact not only on the quality of life and mental health of individuals, but also on productivity and the economy as a whole, as they can lead to the use of health services, absenteeism from work due to illness, reduced productivity, and even permanent disability pensions ^34^. In some European industries, absenteeism rates of over 50% have been reported due to WMSD ^1^. In Brazil approximately 39,000 workers were on leave of absence from work due to WMSD in 2019 alone ^36^.

The spatial distribution of WMSD cases in Brazil is an important issue that requires further analysis to identify the workers and locations most affected by this condition. In this regard, our study found that the Southeast region accounted for more than 50% of all WMSD notifications throughout the time series, which is consistent with previous research. This region is recognized as the largest industrial and economic center in Brazil, with greater access to health services, which could explain the higher number of reported WMSD cases.

The states of São Paulo, Rio de Janeiro, and Minas Gerais are located in the Southeast region. Together, these states comprise Brazil’s largest economic center, with a diversity of economic activities, relevant market shares, and the largest qualified workforce in the country ^5^. The economic production in the Southeast region, particularly in the aforementioned states, reinforces the findings of this study, which show that the region has the highest number of WMSD cases.

The North region, characterized by its main economic activities related to mineral and plant extractives, was the region with the lowest number of WMSD notifications. This finding may be related to the high proportion of informal workers in the region. This fact could potentially conceal actual WMSD cases, compromising active epidemiological surveillance of workers’ health ^5^.

Unlike the other regions, which showed an upward temporal trend, the Northeast region showed a stable trend. This observation may be related to the considerable proportion of informal workers and the inadequate access to health services in the region.

Despite recent improvements in Brazil’s information systems, health issues are still underreported, and even when reported, important data such as patient demographics, clinical outcomes, and mode of entry are often poorly recorded. Therefore, it is important to highlight some limitations of this study, as ecological studies with secondary data cannot establish causal relationships.

Furthermore, we believe that there was significant underreporting in the early years of this time series. This is due to differences in the capacity of local surveillance systems and in the training of professionals. It should also be noted that our study was unable to represent the situation of informal workers, as access to workers’ health policies and services is still incipient in Brazil.

Moreover, the application of these tools in the study of WMSD is still incipient, despite the potential to provide important information for the design and implementation of effective interventions to reduce the burden of these disorders. Our study contributes to filling this gap by presenting a comprehensive and updated analysis of the spatial distribution and temporal trend of WMSD in Brazil, using a rigorous methodology and reliable data sources.

The limitations of this study are related to the use of secondary data, considering that it implies the use of databases with missing and ignored information. Some of the strengths of this study are the use of data collected since the institution of compulsory notification of WMSD in 2007 and the provision of a general and unprecedented overview of the distribution of WMSD in Brazil. In addition, the study offers hypotheses for the development of future studies focusing on local-regional peculiarities.

## Conclusion

Future studies are needed to further characterize the most prevalent injuries associated with WMSD, possibly contributing to the development of more effective rehabilitation services tailored to the local context. In addition, it is crucial to analyze the factors associated with long-term work absences to understand the true economic impact on social security, and the data should also be analyzed by region, considering the degree of informality and industrialization. Lastly, we strongly recommend conducting additional local surveys to evaluate the structure, processes, and outcomes of workers’ health services.

In conclusion, our study identified an increasing temporal trend in WMSD among women and older adults. Trend analysis by region showed a significant increase in all regions except the Northeast, where the trend remained stable. Moreover, the spatial distribution of WMSD showed heterogeneity, with high-risk spatial and space-time clusters in the Brazilian region with the highest level of industrial and economic development. Our findings highlight the need to implement intersectoral surveillance policies, inspect working conditions, and invest in prevention and promotion of workers’ health.
