# Supplementary material for: Space-time analysis of work-related musculoskeletal disorders in Brazil: an ecological study
Source: Cad Saude Publica. 2024 Jul 22;40(7):e00141823. doi: 10.1590/0102-311XEN141823 (PMC11286264; doi:10.1590/0102-311XEN141823)
Supplement: Supplementary file 1 [file 1678-4464-csp-40-07-EN141823-s.pdf]

SUPPLEMENTARY MATERIAL

Table S1 Description of study variables.

Classification			Parameter	Description	Analysis
Dependent			Incidence rate (per 100,000 working age population)	Calculated at the municipality and regional levels	Time trends<br>Spatial cluster<br>Spatiotemporal cluster
Independent	Explanatory	Demographic	Year of occurrence	2007 to 2019	Time trends
			Region of residence	North, Northeast, South, Southeast, and Central-West	Descriptive epidemiological characterization
			Sex	Male and female	
			Age group (years)	≤ 15, 15-24, 25-44, 45-59, and ≥ 60	
			Ethnicity/Skin color	White and non-white	
			Residence zone	Rural, urban, and periurban	
			Level of education	Basic education (< 12 years)<br>Higher education (> 12 years)	
			Case type	New case, relapse, and transference	
			Clinical outcome	Cure, abandonment, death, and transference	
		Clinical	Comorbidities	Hypertension<br>Diabetes<br>Mental disorders	
			Clinical manifestations	Sensitivity alterations<br>Limited range of motion<br>Muscular weakness<br>Decreased movement<br>Phlogistic signs<br>Pain<br>Limitation for activities<br>Absence for treatment	
